# Assessment on the prevalence and risk factors of gastrointestinal parasites on schoolchildren at Bochesa Elementary School, around Lake Zwai, Ethiopia

**DOI:** 10.1186/s13104-019-4446-2

**Published:** 2019-07-15

**Authors:** Ayalew Sisay, Brook Lemma

**Affiliations:** 1 Department of Biology, College of Natural Science, Debre Markos University, P. O. Box 269, Debre Markos, Ethiopia; 2 Department of Zoology, College of Natural Science, Addis Ababa University, P. O. Box 1176, Addis Ababa, Ethiopia

**Keywords:** Children, Lake Zwai, Gastrointestinal, Parasites, Wetlands

## Abstract

**Objectives:**

This study was aimed to assess the prevalence and risk factors of gastrointestinal parasites on schoolchildren at Bochesa Elementary School around Lake Zwai, Ethiopia. Cross-sectional study was conducted on 384 schoolchildren in May 2016. The gastrointestinal parasites were examined with wet mount and formol-ether concentration techniques. Chi-square (χ^2^) test was used to evaluate the association between categorical variables and infection prevalence. Binary logistic regression on SPSS version 21 was used, values were considered significant when the *p*-value was less than 0.05.

**Results:**

The overall prevalence of gastrointestinal parasites was 22.6%. Males, 54 (14.1%) were more infected than females, 32 (8.3%), and 1–4 grade category, 64 (16.7%) were more infected than 5–8 grade category, 22 (5.7%). Age groups of 7–14, 78 (20.3%) were also more infected than > 15, 8 (2.1%); however, the variation was not significant (*p *> 0.05). In this study, parasitic coinfection was common; however, single gastrointestinal parasites were more dominant. The overall rate of gastrointestinal parasites shows that the environmental conditions where students pass their times are conducive to water-related diseases. Health education on personal and environmental hygiene keeping should be given to schoolchildren and safe wetland playing grounds should be prepared.

## Introduction

Many civilizations have been built on the basis of wetland resources [1]; this indicates that wetlands and people are ultimately interdependent with each other [2, 3]. In developing countries, the livelihoods of people are intimately linked with wetlands and these interactions lead to the emergence of gastrointestinal parasitic infections [4].

Faecal-oral contact of infective parasitic stages is important route to human infection by water-related gastrointestinal parasites [5, 6]. Factors like eating raw vegetables, lack of hygiene, unsafe drinking water, lack of toilet facilities, walking barefoot, fishing, irrigation, swimming, playing with moist soil and open defecation may increase the prevalence of gastrointestinal parasites [7] of wetland areas.

The prevalence of gastrointestinal parasites varies from region to region [7]. Prevalence of gastrointestinal parasites has been studied in different countries including Ethiopia [7, 8]. It was found out that these diseases are more prevalent on school age children [9–11]. Hence, the present study was aimed at assessing the prevalence and risk factors of gastrointestinal parasitic infections on schoolchildren at a school located in close proximity to the wetlands of Lake Zwai.

## Main text

### Methods

#### Study setting and period

Cross-sectional study was conducted in May 2016 on Bochesa Elementary schoolchildren around Lake Zwai, which is located in the Southwestern direction of lake Zwai, about 167 km South of Addis Ababa, which is the capital of Ethiopia. The school is situated at an altitude of 1636 m above sea level, which is similar to the lake. Students of the school are living under poor socio-economic status and with no adequate safe water supply in their village; and their parents lead their life by cultivation of maize and vegetables using lake water for irrigation and rearing of livestock.

#### Sample size and sampling techniques

The desired sample size (n) required for the assessment of gastrointestinal parasites and associated risk factors was estimated using standard formula [12].

$${\text{n}} = \frac{{\left( {z^{2} p\left( {1 - p} \right)} \right)}}{{d^{2} }},$$

where n is sample size, z is 1.96, which is z statistics level of confidence, d = 0.05 (absolute precision), and p = expected prevalence of the area.

Since the prevalence of gastrointestinal parasites is not known for this study area on schoolchildren, *p*-value was taken to be 50%. A 95% confidence interval (z) and a 5% margin of error (d) was used. Therefore, 384 (192 males and 192 were females) students from 7 to 25 years were involved.

#### Laboratory investigations

Stool samples were collected using applicator sticks in a labeled plastic container.

About 5 g of fresh fecal samples were collected from each students and placed in separate labeled clean plastic stool containers. A small portion of about 2 g sample from each student was examined using wet mount and the remaining about 3 g sample was examined using formol-ether concentration technique [13]. The overall presence of gastrointestinal parasites was confirmed when observed by any of the methods used.

#### Questionnaire administration

A questionnaire was developed in English and translated into Afan Oromo (the local language) to collect socio demographic data, environmental factors and behavioral habits.

#### Statistical analysis

The prevalence of infections was reported in proportions. Chi-square (χ^2^) test was used to evaluate the association between categorical variables and prevalence. For identification of determinant factors, Binary logistic regression was used, and finally the association between independent variables and dependent variables were described on the basis of odd ratio (OR) with 95% confidence interval (CI). Crude OR was estimated by univariate regression analysis and adjusted OR was then estimated by multivariate logistic regression analysis. Values were considered statistically significant when the *p*-value was less than 0.05.

### Results

The overall prevalence of gastrointestinal parasites infection was 22.4% (95% CI 18–26.3%). Different types of parasites like cestodes, nematodes and protozoans with a value of 37 (9.6%), 40 (10.4%) and 29 (7.6%) were identified, respectively from Bochesa Elementary School students. Of these eight species identified, *Hymenolepis nana* was the predominant parasites detected on 34 (8.9%), and least prevalence was recorded on *Taenia* spp. with a value of 3 (0.8%) (Table 1).

**Table 1:** Different species of gastrointestinal parasites and their prevalence on Bochesa Elementary School

Parasites	Species obtained	Frequency	Prevalence (%)
Nematodes	*A. lumbricoides*	17	4.43
	*T. trichuria*	8	2.08
	*S. stercoralis*	8	2.08
	Hookworm spp.	14	3.65
Cestodes	*H. nana*	34	8.85
	Taenia spp.	3	0.78
Protozoa	*E. histolytica/dispar*	20	5.21
	*G. lamblia*	18	4.69

The prevalence of gastrointestinal parasites was statistically significant (χ^2^ = 7.252; *p* = 0.005 and χ^2^ = 6.177; *p* = 0.008) between sex and grade categories, respectively; whereas insignificant (*p *> 0.05) variations was observed on the prevalence of gastrointestinal parasites between age groups.

**Fig. 1:**
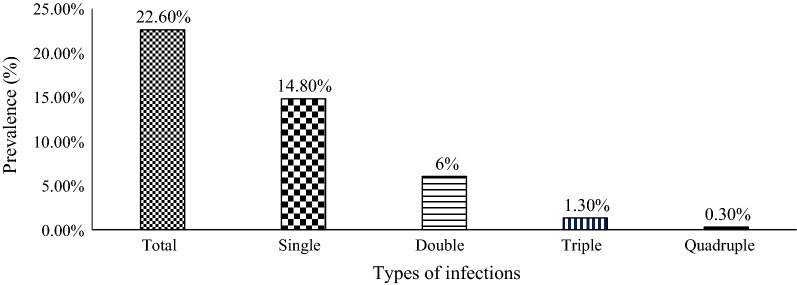
Prevalence of single and mixed parasitic infections among Bochesa Elementary School students

Of these 86 (22.4%) positive cases, infection with single parasite was more dominant (14.8%) than mixed infections, in which double, triple and quadruple parasitic infections with a value of 6.0%, 1.3% and 0.3%, respectively (Fig. 1). The prevalence of single parasitic infection was statistically significant (χ^2^ = 4.635; *p* = 0.022) between se; whereas insignificant (*p* > 0.05) variations were observed with age and grade category.

#### Potential risk factors associated with gastrointestinal parasites

Being male increases the risk of gastrointestinal parasite infection 7.79-folds than being female (AOR: 7.799: 95% CI 1.948–31.222). Students who were from 5 to 8 grade category decreases the risk of gastrointestinal parasite infection by 69.3% than students from 1 to 4 grade category (AOR: 0.307: CI 95% 0.110–0.855). Students who had fishing habits were 3.027 times more likely to be infected by gastrointestinal parasites than their counter parts (AOR: 3.027: CI 95% 1.117–8.206). Students who had raw meat-eating habits were 4.710 times more likely to be infected by gastrointestinal parasites than students who didn’t eat raw meat (AOR: 4.710: CI 95% 1.626–13.643). Similarly, students who had raw vegetable eating habit were 2.8 times more likely to be infected by gastrointestinal parasites than their counter parts (AOR: 2.800: CI 95% 1.126–6.961). Students who had habits of playing with moist soil were 8.571-folds increases the risk of gastrointestinal parasites than students who didn’t play (AOR: 8.571: CI 95% 3.884–18.913). Similarly, students who had sometimes hand washing habit before meals were 4.961-folds increased the risk of gastrointestinal parasites than students who washed their hands before meals (AOR: 4.961: CI 95% 1.773–13.879). Students who practiced open defecation showed increased the risk of gastrointestinal parasite infections with a rate of 6.12 times more than students who used latrines (AOR: 6.118: CI 95% 2.414–15.510) (Table 2).

**Table 2:** Binary logistic regression analysis for factors potentially associated with gastro intestinal parasite infection among Bochesa Elementary Schoolchildren

Risk factors	Category	Positive (%)	Negative (%)	Adjusted OR (95% CI)
Sex	Male	51	141	7.779* (1.948–31.222)
	Female	32	160	
Grade category	1–4	61	181	0.307* (0.110–0.855)
	5–8	22	120	
Fishing habit	Yes	53	117	3.027* (1.117–8.206)
	No	30	184	
Raw meat-eating	Yes	63	165	4.710* (1.626–13.643)
	No	20	136	
Playing with moist soil	Yes	36	34	8.571* (3.884–18.913)
	No	47	267	
Water for bathing	Lake water	79	255	2.125 (0.720–6.274)
	Ground water	4	46	
Raw vegetable eating	Yes	47	88	2.800* (1.126–6.961)
	No	36	213	
Hand washing	Sometimes	27	13	4.961* (1.773–13.879)
	Yes	56	288	
Finger nail trimming	Yes	32	200	
	No	51	101	1.869 (0.801–4.361)
Area of defecation	Latrine	9	151	
	Open defecation	74	150	6.118* (2.414–15.510)
Shoe wearing habit	Yes	27	190	2.109 (0.852–5.220)
	No	56	111	

*Significant association

### Discussion

The present study revealed the occurrence of eight species of gastrointestinal parasite on schoolchildren. The overall prevalence (22.4%) of gastrointestinal parasites observed in this study is comparable with reported cases of Ethiopia [14] and Angola [15].

The present result is also lower when compared with other earlier reports from different parts of the country [7, 9, 16, 17], and outside (Turkey [18] and Egypt [19]). The probable reason for this variation might be attributed to the variation in environmental and living conditions of the study participants.

The occurrence of gastrointestinal helminth parasitic infections among students of Bochesa Elementary School found in Zwai wetlands is an indication of faecal contamination of soil, improper utilization of latrines or absence of them and poor personal hygiene in the area. Similar finding was reported in Zwai Town on pregnant woman [20].

In this study, *H. nana* (8.9%) was the dominant parasite. Similar findings were reported inside the country [21, 22]. The observed prevalence of 8.6% for *H. nana* in this study was in agreement with different researchers inside the country [8, 23, 24]; and in Burkina Faso [25]. This result was also relatively higher compared to other studies in different areas of the country [26–31]. This difference might be due to the variation in environmental and living conditions of the study participants.

Higher prevalence of gastrointestinal parasites on males in this study was supported by different researchers inside the country [8, 12, 22, 23] and elsewhere [32, 33]. The probable reason for this variation might be due to more involvement of males in outdoor activities. These activities sometimes carried out bare-footed and this situation predisposing males to infections.

Similarly, higher prevalence of gastrointestinal parasites on lower age groups was in agreement with other reports inside the country [29, 34–36], and Bangalore [37]. The probable reason for this difference might be due to frequent contact and the habit of inserting soil contaminated fingers to their mouth and less awareness of hand washing practices may increase the chance of acquiring gastrointestinal parasites in lower age groups.

Higher prevalence of gastrointestinal parasites on 1–4 grade category was also inline with other studies in Ethiopia [21, 29, 34]. The probable reason for this variation might be due to awareness created to improve personal hygiene in 5–8 grades. The present study revealed that the existence of parasitic co-infection in the wetland of Lake Zwai in Bochesa Elementary schoolchildren. In line with this findings, parasitic co-infections were reported inside the country [7, 9, 16, 29, 38].

The possible reason for the existence of parasitic co-infections in the study area might be associated with Zwai wetland, which is found in front of their residences to make frequent contact for swimming, bathing, fishing and irrigation purpose in the lake and the wetlands around it. In this study, single parasitic infections were more common than parasitic co-infections. These results were comparable with studies conducted elsewhere in Ethiopia [9, 16, 17, 21, 30].

The present study shows that the likelihood of acquiring gastrointestinal parasites in males was 7.79 times higher than in females. In accordance with these findings, agricultural and fishing areas at the lake borders and irrigation canals are common defecation places by males during working times, facilitating the transmission of gastrointestinal parasites would be facilitated. Similar findings were reported in studies conducted elsewhere in Ethiopia [14, 22].

Students having raw-meat eating habits were 4.71 times more likely to be infected by gastrointestinal parasites such as *Taenia* spp. than students who had no raw-meat eating habits. Recently, the custom of eating raw-meat has grown worldwide in ethnic groups where eating raw-meat was not previously common [5].

Students who had raw vegetable-eating habit were 2.80 times more likely to acquire gastrointestinal parasites than students who had no raw vegetable-eating habits. This finding was supported by studies in Ethiopia [39–42].

Students who played with moist soil were 8.57 times more likely to be infected by gastrointestinal parasites than students who had no such habits. This might be due to the fact that moist soil creates an environment conducive for a high prevalence of intestinal parasites [43, 44].

Students who have practiced open defecation were 6.12 times more likely to acquire gastrointestinal parasites than those who used latrines. Similar findings were reported [8, 27, 41].

### Conclusion

The study revealed that the wetland of Lake Zwai are conducive for the survival of water-related gastrointestinal parasites as observed in the schoolchildren of the area. As the weather condition of Zwai wetlands is hot and humid coupled with wet soil is a suitable environment for the occurrence of infective stages of gastrointestinal parasites. These disposing factors to the disease is further increased due to the poor economic status of the community of the area and the nature of students who do not see the risks of infection by gastrointestinal parasites that are out there.

## Limitations

We used only wet mount and formol-ether concentration technique to assess the prevalence of gastrointestinal parasites in the collected samples.

## Data Availability

The data used and analyzed in this study are available from the corresponding author on reasonable request.
